# A Systematic Analysis of Biological, Sociodemographic, Psychosocial, and Lifestyle Factors Contributing to Work Ability Across the Working Life Span: Cross-sectional Study

**DOI:** 10.2196/40818

**Published:** 2023-05-19

**Authors:** Patrick D Gajewski, Jennifer A Rieker, Georgios Athanassiou, Peter Bröde, Maren Claus, Klaus Golka, Jan G Hengstler, Thomas Kleinsorge, Michael A Nitsche, Jörg Reinders, Anita Tisch, Carsten Watzl, Edmund Wascher, Stephan Getzmann

**Affiliations:** 1 Leibniz Research Centre for Working Environment and Human Factors (IfADo) at the Technical University of Dortmund Dortmund Germany; 2 Department of Basic Psychology II Universidad Nacional de Educación a Distancia Madrid Spain; 3 Maritime and Logistic Studies Jade University of Applied Sciences Elsfleth Germany; 4 Federal Institute for Occupational Safety and Health (BAuA) Dortmund Germany

**Keywords:** aging, cognitive aging, cardiovascular system, immunology, lifestyle, metabolism, occupational safety and health, public health, psychosocial stress, work ability, mobile phone

## Abstract

**Background:**

As employees age, their physical and mental abilities decline and work ability decreases, enhancing the risk for long-term sick leave or even premature retirement. However, the relative impact of biological and environmental determinants on work ability with increasing age is poorly understood in terms of their complexity.

**Objective:**

Previous research has shown relationships between work ability and job and individual resources, as well as specific demographic and lifestyle-related variables. However, other potentially important predictors of work ability remain unexplored, such as personality traits and biological determinants, including cardiovascular, metabolic, immunological, and cognitive abilities or psychosocial factors. Our aim was to systematically evaluate a wide range of factors to extract the most crucial predictors of low and high work ability across the working life span.

**Methods:**

As part of the *Dortmund Vital Study*, 494 participants from different occupational sectors, aged between 20 and 69 years, completed the Work Ability Index (WAI) assessing employee’s mental and physical resources. A total of 30 sociodemographic variables were grouped into 4 categories (social relationships, nutrition and stimulants, education and lifestyle, and work related), and 80 biological and environmental variables were grouped into 8 domains (anthropometric, cardiovascular, metabolic, immunologic, personality, cognitive, stress related, and quality of life) and have been related to the WAI.

**Results:**

Using the analyses, we extracted important sociodemographic factors influencing work ability, such as education, social activities, or sleep quality, and identified age-dependent and age-independent determinants of work ability. Regression models explained up to 52% of the WAI variance. Negative predictors of work ability were chronological and immunological age, immunological inefficiency, BMI, neuroticism, psychosocial stress, emotional exhaustion, demands from work, daily cognitive failures, subclinical depression, and burnout symptoms. Positive predictors were maximum heart rate during ergometry, normal blood pressure, hemoglobin and monocyte concentration, weekly physical activity, commitment to the company, pressure to succeed, and good quality of life.

**Conclusions:**

The identified biological and environmental risk factors allowed us to evaluate work ability in its complexity. Policy makers, employers, and occupational safety and health personnel should consider the modifiable risk factors we identified to promote healthy aging at work through focused physical, dietary, cognitive, and stress-reduced preventive programs, in addition to well-balanced working conditions. This may also increase the quality of life, commitment to the job, and motivation to succeed, which are important factors to maintain or even enhance work ability in the aging workforce and to prevent early retirement.

**Trial Registration:**

ClinicalTrials.gov NCT05155397; https://clinicaltrials.gov/ct2/show/NCT05155397

**International Registered Report Identifier (IRRID):**

RR2-10.2196/32352

## Introduction

### Background

In Western and East Asian societies, the average chronological age and employment rate of older workers is rising. The reasons for this are the need to work longer owing to higher life expectancy, a shortage of qualified young professionals, and the safeguarding of social security and pension systems. For example, in North America, Europe, and the Asia-Pacific Region, employment rates for people aged 55 to 64 years continuously increased between 2011 and 2021 from 57.7% to 62.7% in Canada, to a lesser extent from 59.9% to 61.8% in the United States, from 61.1% to 65.4% in Australia, from 45.1% to 60.5% in the European Union, and exemplarily from 59.1% to 71.8% in Germany [1]. At the same time, aged workers are facing new challenges in modern work, such as the introduction of new technologies, increasing complexity and density of work, new spatial work concepts, documentation obligations, increase in electronic communication, need for qualification, and permanent accessibility [2,3]. However, as workers age, their physical and mental abilities decline, and work-related illnesses increase, which may lead to occupational accidents, frequent or prolonged absenteeism, involuntary withdrawal from active working, or early retirement [4-8]. Therefore, it is crucial to develop different methods that optimally match individually available capabilities with work-related demands to prevent such scenarios and improve the employability of older workers [9-11].

The relationship between individual resources and work-related demands is labeled work ability. The concept work ability was developed in the early 1980s in the Finnish Institute of Occupational Health and refers to an individual’s ability to perform the required work [6]. Work ability is a multifactorial concept and results from the balanced interaction between job demands and characteristics (mental, physical, emotional, social, and organizational), individual skills and abilities of employees, and their health status and subjective self-perception (in terms of values, attitudes, and motivation) in a given work environment [5].

The most frequently used instrument for evaluating work ability is the Work Ability Index (WAI) [12]. It reflects current and future subjective appraisal of employees with respect to the interplay of physical and psychological work demands, individual health status, and physical and mental capacities. It considers specific psychosocial and physical factors associated with performing a given type of work as well as the employee’s mental and physical resources and health condition [13].

As work ability is a complex construct, the relative impact of potential determinants must be assessed to develop interventions for the prevention of occupational risks and maintenance of productivity and health as constituents of sustainable work. Several previous studies, reviews, and meta-analyses have examined the major factors contributing to work ability, such as age, demographic variables, BMI, physical activity, lifestyle factors, and physical and psychosocial work demands [14-16]. However, other important determinants such as personality traits, cognitive abilities, and biological parameters have rarely been considered. This study aimed to close this gap by including unexplored but potentially important factors contributing to work ability.

### This Study

This exploratory study was conducted as part of the *Dortmund Vital Study* [17] (ClinicalTrials.gov NCT05155397), a multifactorial, longitudinal study with follow-up measures to investigate sources of interindividual differences in physical, metabolic, immunological, and cognitive functioning across the working life span. The cross-sectional part of the study presents an analysis of the baseline measurements.

The novelty of this study is that, in addition to several sociodemographic factors, individual and work-related determinants were grouped into 8 theoretically founded domains that were further analyzed in relation to work ability: anthropometric, cardiovascular, metabolic, immunological, personality, cognitive, quality of life, and stress-related factors, each represented by several parameters. Some of the analyzed domains were reported for the first time in relation to work ability. To assess their impact on work ability as a function of age, the sample was further divided into 3 age groups, comprising young, middle-aged, and older participants. The aim of the analytical strategy was to track potential associations between biological and environmental determinants and work ability across the working life span, as the impact of these relationships may be modulated by age and increasing personal and work experiences.

Next, critical predictors of work ability were extracted for each domain through a series of standard multiple linear regression analyses and included in a final regression model to identify the most essential determinants of work ability.

## Methods

### Study Population

Participants of the *Dortmund Vital Study* were adults aged between 20 and 70 years, with ages distributed almost evenly across decades. The sample and further details such as eligibility criteria, methods, and procedures are described in detail in the study protocol [17]. The participants were generally healthy, with older participants often taking typical medications such as antihypertensives, blood thinners, hormones, and cholesterol reducers. From a total of 588 participants, we selected participants who were currently employed in part-time or full-time jobs and had completed the long version of the WAI. These participants worked in the following main common employment sectors: industry (eg, engineering, mechanics, shop fitters, mechatronics, or logistics), service (nurse, social worker, physician assistance, police officer, fire service, hairdressing, waiter, seller, bank assistance, or laboratory assistance), education (eg, teacher, working student, childcare work, or instructor), and craft (automotive mechanic, plumber, electrician, or builder). No restrictions were imposed regarding specific occupational groups.

The final number of participants in this study was N=494 (317/494, 64.2% females). The mean age was 42.7 (SD 12.7; range 20-69) years. The sample was divided into terciles according to age into 3 groups: young adults (177/494, 35.8%; mean 28.2, SD 4.2; range 20-35 years), middle-aged adults (182/494, 36.8%; mean 45.3, SD 4.9; range 36-52 years), and older adults (134/494, 27.1%; mean 58.1, SD 3.9; range 53-69 years). Data were collected between 2016 and 2020.

### Primary Outcome

As a primary outcome, the total WAI score was calculated by summing the scores of the 7 WAI categories, computed according to the manual. The WAI can range between 7 and 49 points (Table 1) and is classified into the following categories: poor (score 7-27), moderate (score 28-36), good (score 37-43), and excellent (score 44-49) work ability.

**Table 1:** Mean scores with SDs of the analyzed cohort in the 7 Work Ability Index (WAI) categories, the possible range of scores, and the total score^a^.

WAI category	Values, mean (SD)	Values, range
Current work ability compared with the lifetime best	7.99 (1.6)	0-10
Work ability in relation to the demands of the job	8.38 (1.4)	2-10
Current diseases diagnosed by a physician	3.42 (2.2)	1-7
Estimated work impairment due to diseases	5.53 (0.9)	1-6
Sick leave during the past year (12 months)	4.09 (0.9)	1-5
Own prognosis of work ability 2 years from now	6.85 (0.7)	1-7
Mental resources	3.18 (0.7)	0-4

^a^Total score: 39.16 (SD 5.6; range 7-49).

### Predictors

The predictors were grouped into 8 domains: anthropometric, cardiovascular, metabolic, immunological, personality, cognitive, stress related, and quality of life. The details of all measures are described in Multimedia Appendix 1 and in the study protocol [17].

### Statistical Analysis

Descriptive statistics, frequencies, and distributions were calculated for all quantitative variables. The data analysis comprised the following steps. First, the qualitative variables of the sociodemographic questionnaire were categorized into 4 groups (family and social relationships, nutrition and stimulants, education and lifestyle, and work-related aspects), which were then analyzed in relation to the total WAI score. Summary statistics, including mean and SD, were provided for all sociodemographic variables. Independent sample *t* tests (2-tailed) or 1-way ANOVAs were conducted to compare the total WAI scores between the categories of each sociodemographic variable.

Second, for the whole sample and for each age group, Pearson correlations were computed for each of the 8 domains to determine the relationship between the biological and environmental markers on the one hand and the WAI score, on the other. No adjustment of the α level was applied to prevent type 2 errors, that is, not detecting possibly relevant associations. This is a common and consented strategy in epidemiological and exploratory studies, in which the information obtained from the data prevails over the number of tests conducted [18-21].

Third, a series of multiple regression analyses was conducted to identify predictors of work ability for each domain separately. In each regression model, the chronological age was included. Missing data were handled with listwise deletion. Predictors with a large amount of missing data (approximately of 50% hair cortisol, ammonia, and toxoplasmosis status) were excluded from the analysis. The data were controlled for multicollinearity, normality, linearity, and independence of residuals. Data were also assessed for multivariate outliers using the Mahalanobis Distance Test [22]. Multivariate outliers were identified by calculating the probability of the cumulative distribution functions of the chi-square distribution and were removed when the probability was *P*<.001. The Mahalanobis Distance Test requires a complete number of cells to be computed. Depending on the domain, between 1 and 22 multivariate outliers were discarded. Multicollinearity was evaluated using the variance inflation factor (VIF). Predictors with VIF>3 were excluded [23]. In addition, if the VIF was close to 3, we analyzed the correlation coefficients and removed those with *r*>.07.

The series of regression analyses was conducted separately to extract the most substantial predictors of work ability within each domain, which were then submitted to the final regression analysis that included these predictors. This strategy allows for easier detection of multicollinearity because of similar measures or overlapping predictors within each domain. Furthermore, we ran a series of standard multiple linear regressions rather than a single regression analysis with all predictors included simultaneously to reduce the impact of missing data. As we did not impute missing data and instead used standard listwise exclusion, the number of excluded cases would be too large (approximately 40%) to draw reliable conclusions. The most common reasons for missing data were incompletely filled in tests or questionnaires, insufficient biological samples, technical problems, or problems in completing bicycle ergometry.

In the last step, we performed a final regression analysis with the variables found to be substantially significant in the series of regression analyses in the previous step. A backward elimination model was used, in which independent variables were first entered into the equation, and each variable was deleted individually if it did not contribute to the regression model. Data were analyzed using the SPSS (version 28; IBM Corp).

### Ethics Approval, Informed Consent, and Participation

The study was approved by the local ethics committee of the Leibniz Association at IfADo in October 2015 (approval number: A93-1), in accordance with the Declaration of Helsinki. All participants were informed of the scope of the study and provided written informed consent before any study protocol commenced. The study data were pseudonymized during the course of the study to prevent personal identification [17]. The participants received €160 (US $175) compensation for complete testing, which was held over 2 days.

## Results

### WAI Scores

Overall, the WAI yielded a mean score of 39.16, indicating good work ability in the present cohort (Table 1).

### Sample Characteristics and Lifestyle Variables

Groups of variables as a function of the WAI are presented in Tables 2-5. Table 2 presents sociodemographic variables, family and social relationships, Table 3 presents nutrition and stimulants, Table 4 presents education and free time activities, and Table 5 presents work-related aspects. Marital status, raising underage children, living situation, care of relatives, nutrition, or the use of stimulants—such as smoking or alcohol consumption—were not related to work ability. Social components, such as the number of close friends and the frequency of friendship meetings, were associated with enhanced work ability. A pet in the family was associated with lower work ability. Education and frequency of using a foreign language were related to higher work ability. Individuals with elementary and high school degrees scored lower in work ability than did those with secondary or higher education degrees. Furthermore, a negative relationship was observed between sleep quality and work ability. Although the frequency of computer or smartphone use was not associated with work ability, individuals who watched >3 hours of television per day had lower scores in work ability. Work amount (ie, full time vs part time), type of contract (permanent vs temporary), and commuting to work did not substantially relate to work ability. However, individuals employed in education demonstrated higher work ability than those employed in industry or services, and individuals with repetitive job characteristics had a substantially lower WAI score than those with flexible job characteristics.

**Table 2:** Sociodemographic, family, and social characteristics as a function of the Work Ability Index (WAI) score (N=494).

Characteristics				Values, n (%)		WAI, mean (SD)		Test statistics			*P* value	
**Age groups**									*F_2,493_=15.5^a^*			*.001*
	Young adults		174 (35.2)		41.0 (4.8)							
	Middle-aged adults		185 (37.4)		38.2 (6.2)							
	Older adults		135 (27.3)		38.0 (5.3)							
**Family and social relationships**												
	**Marital status^b^**							*F*_4,490_=0.39			.82	
		Single	37 (7.5)		40.2 (5.0)							
		Married	185 (37.7)		39.1 (5.9)							
		Partnership	227 (46.2)		39.0 (5.7)							
		Divorced	36 (7.3)		39.4 (4.5)							
		Widowed	6 (1.2)		39.3 (4.0)							
	**Living situation^c^**							*F*_4,455_=1.90			.11	
		Alone	105 (23)		38.9 (6.8)							
		With partner	138 (30.3)		39.5 (4.8)							
		With family	160 (35.1)		38.7 (5.6)							
		With parents	10 (2.2)		41.1 (5.4)							
		With other persons	43 (9.4)		41.0 (4.5)							
	**Children**							t_492_=1.25			.21	
		No	332 (67.2)		39.4 (5.7)							
		Yes	162 (32.8)		38.7 (5.5)							
	**Care of relatives^d^**							t_469_=1.44			.15	
		No	426 (86.2)		39.2 (5.7)							
		Yes	45 (9.1)		37.9 (5.7)							
	**Family pet**							*t_492_=3.15*			*.002*	
		No	385 (77.9)		39.6 (5.3)							
		Yes	109 (22.1)		37.6 (6.6)							
	**Number of close friends**							*F_4,470_=2.40*			*.048*	
		1-3	98 (20.8)		37.7 (6.3)							
		4-7	227 (48.2)		39.1 (5.6)							
		8-10	90 (19.1)		39.7 (5.2)							
		>10	51 (10.8)		40.3 (5.0)							
	**Frequency of friendship meetings**							*F_4,490_=4.45*			*.002*	
		1 or 2 per month	141 (28.7)		37.6 (6.2)							
		1 per week	144 (29.3)		39.1 (5.5)							
		2 per week	113 (23)		39.9 (4.9)							
		3 per week	62 (12.6)		40.5 (5.0)							
		>3 per week	31 (6.4)		40.5 (5.8)							

^a^Italicized values are those with significant results.

^b^6 (1.2%) missing data.

^c^41 (8.2%) missing data.

^d^23 (4.6%) missing data.

**Table 3:** Nutritional characteristics and use of stimulants as a function of the Work Ability Index (WAI) score (N=494).

Characteristics			Values, n (%)	WAI, mean (SD)	Test statistics		*P* value	
**Nutrition and stimulants**								
	**Frequency of *fruit* consumption**					*F*_4,492_=1.35		.25
		Several times a day	80 (16.2)	39.2 (5.7)				
		Daily	184 (37.3)	39.8 (5.7)				
		1-4 per week	206 (41.8)	38.7 (5.5)				
		1 per month	19 (3.9)	37.5 (6.3)				
		Never	4 (0.8)	37.7 (5.9)				
	**Frequency of *vegetable* consumption**					*F*_3,492_=2.12		.10
		Several times a day	70 (14.2)	40.2 (5.5)				
		Daily	222 (45)	39.5 (5.5)				
		1-4 per week	195 (39.6)	38.5 (5.7)				
		1 per month	6 (1.2)	38.0 (5.8)				
		Never	0 (0)	N/A^a^				
	**Frequency of *wholemeal* consumption**					*F*_4,490_=0.390		.82
		Several times a day	37 (7.5)	40.2 (5.0)				
		Daily	185 (37.7)	39.1 (5.9)				
		1-4 per week	227 (46.2)	39.0 (5.7)				
		1 per month	36 (7.3)	39.4 (4.0)				
		Never	6 (1.3)	39.3 (4.0)				
	**Frequency of *meat* consumption**					*F*_4,491_=0.345		.85
		Several times a day	93 (18.9)	39.4 (5.5)				
		Daily	113 (23)	39.5 (5.9)				
		1-4 per week	203 (41.3)	38.9 (5.8)				
		1 per month	28 (5.7)	38.7 (5.8)				
		Never	55 (11.1)	39.1 (6.1)				
	**Frequency of *fish* consumption**					*F*_4,493_=1.74		.14
		Several times a day	2 (0.4)	46 (0)				
		Daily	4 (0.8)	41.7 (2.2)				
		1-4 per week	230 (46.6)	39.5 (5.3)				
		1 per month	197 (39.9)	38.5 (6.0)				
		Never	61 (12.3)	39.2 (5.4)				
	**Frequency of *fast-food* consumption**					*F*_4,492_=1.09		.36
		Several times a day	1 (0.2)	40 (0)				
		Daily	3 (0.6)	38.6 (1.5)				
		1-4 per week	123 (24.9)	39.1 (5.8)				
		1 per month	309 (62.7)	38.9 (5.6)				
		Never	57 (11.6)	40.6 (5.4)				
	**Frequency of *alcohol* consumption**					*F*_3,492_=0.902		.44
		Daily	24 (4.9)	38.1 (6.0)				
		1-3 per week	174 (35.3)	38.8 (5.6)				
		1-3 per month	228 (46.2)	39.3 (5.6)				
		Never	67 (13.6)	39.8 (5.4)				
	**Frequency of *smoking***					*F*_4,491_=0.148		.95
		>20 per day	9 (1.8)	37.8 (6.6)				
		11-20 per day	23 (4.7)	39.5 (6.0)				
		1-10 per day	45 (9.2)	39.1 (6.8)				
		No more	103 (20.9)	39.4 (5.0)				
		Never	312 (63.4)	39.1 (5.6)				

^a^N/A: not applicable.

**Table 4:** Education, sleep quality and free time activities as a function of the Work Ability Index (WAI) score (N=494).

Characteristics		Values, n (%)		WAI, mean (SD)		Test statistics			*P* value	
**Education**							*F_3,491_=9.59* ^a^			*<.001*
	University degree	215 (43.7)	37.8 (6.6)							
	High school diploma	153 (31.1)	39.5 (6.0)							
	Secondary	102 (20.7)	39.1 (6.8)							
	Primary	22 (4.5)	37.7 (5.8)							
**Frequency of using a foreign language**							*F_3,491_=5.53*			*<.001*
	Often	143 (29.1)	40.7 (4.7)							
	Sometimes	166 (33.7)	38.7 (5.4)							
	Rarely	145 (29.5)	38.8 (6.1)							
	Never	38 (7.7)	36.4 (6.7)							
**Sleep quality**							*F_3,493_=22.66*			*<.001*
	Always good	44 (8.9)	41.9 (4.4)							
	Mostly good	304 (61.5)	40.1 (4.9)							
	Rarely good	136 (27.6)	36.5 (6.3)							
	Never good	10 (2)	33.2 (5.2)							
**Watching television**							*F_3,288_=7.57*			*<.001*
	Never	207 (41.9)	39.3 (5.9)							
	≤1 hour per day	64 (13)	40.7 (5.0)							
	1-2 hours per day	129 (26.1)	38.2 (5.6)							
	2-3 hours per day	67 (13.5)	40.4 (4.4)							
	>3 hours per day	27 (5.5)	36.0 (6.3)							
**PC use (in free time)**							*F*_3,201_=0.782			.50
	Never	293 (59.3)	39.2 (5.8)							
	1 hour or less per day	95 (19)	38.6 (6.3)							
	1-2 hours per day	64 (13)	39.7 (4.2)							
	2-3 hours per day	26 (5.3)	40.0 (4.8)							
	>3 hours per day	17 (3.4)	38.5 (4.8)							
**Mobile phone use (in free time)^b^**							*F*_3,233_=.052			.98
	Never	261 (52.8)	39.0 (6.0)							
	≤1 hour per day	123 (24.9)	39.5 (5.2)							
	1-2 hours per day	69 (14)	39.2 (5.9)							
	2-3 hours per day	22 (4.5)	39.4 (4.9)							
	>3 hours per day	19 (3.8)	39.2 (4.3)							
**Media general**							*F*_3,404_=1.68			.17
	Never	91 (18.5)	39.2 (6.5)							
	≤1 hour per day	46 (9.3)	38.6 (6.2)							
	1-2 hours per day	82 (16.6)	39.4 (4.2)							
	2-3 hours per day	99 (20)	40.1 (5.4)							
	>3 hours per day	176 (35.6)	38.7 (5.6)							
**Duration of physical activity**							*F_4,493_=3.79*			*.005*
	Never	108 (21.9)	38.0 (6.0)							
	≤1 hour per day	52 (10.5)	39.2 (4.9)							
	1-2 hour per week	99 (20)	38.3 (5.9)							
	2-3 hour per week	66 (13.4)	39.3 (5.6)							
	>3 hours per day	169 (34.2)	40.4 (5.3)							

^a^Italicized values are those with significant results.

^b^The use of PC and mobile phones was surveyed using the question “I prefer to spend my free time with: If yes, how many hours/day?” This does not indicate the total time of PC or mobile phone use but only the time spent in free time, if preferred.

**Table 5:** Work-related parameters of the sample as a function of the Work Ability Index (WAI) score (N=494).

Characteristics		Values, n (%)	WAI, mean (SD)	*Test statistics*		*P* value	
**Work amount^a^**					*F*_4,483_=1.68		.17
	Full position (100%)	219 (45.2)	39.5 (5.6)				
	Three-quarter position (75%)	63 (13)	37.7 (5.3)				
	Half position (50%)	58 (12)	38.8 (4.9)				
	Lesser than 50%	38 (7.9)	38.5 (7.2)				
	Mini job	106 (21.9)	39.7 (5.3)				
**Employment sector^b^**					*F_3,458_=5.23* ^c^		*<.001*
	Industry	42 (8.6)	38.5 (5.7)				
	Service	315 (64.2)	38.6 (6.0)				
	Education	123 (24.9)	41.0 (4.2)				
	Craft	11 (2.2)	39.3 (4.5)				
**Type of work^d^**					*t_460_=2.14*		*.03*
	Repetitive	46 (9.3)	37.6 (6.3)				
	Flexible	416 (84.2)	39.5 (5.5)				
**Shift work^e^**					*F*_3,406_=1.68		.17
	No shift work (day)	347 (81.8)	39.3 (5.5)				
	Shift work without night work	34 (8)	37.0 (6.5)				
	Shift work with night work	38 (9)	39.1 (6.6)				
	Night work only	5 (1.2)	38.4 (11.1)				
**Type of employment contract^f^**					*F*_2,385_=0.878		.48
	Permanent position	269 (69.7)	39.0 (5.7)				
	Temporary position	91 (23.6)	39.7 (6.2)				
	Self-employed	26 (6.7)	40.0 (4.7)				
**Commuting to work^g^**					*F*_3,352_=1.14		.32
	≤1 hour per day	265 (75.1)	38.9 (5.8)				
	1-2 hours per day	71 (20.1)	38.8 (6.0)				
	2-3 hours per day	12 (3.4)	41.1 (4.0)				
	>3 hours per day	5 (1.4)	42.2 (2.9)				

^a^4 (0.8%) missing data.

^b^4 (0.8%) missing data.

^c^Italicized values are those with significant results.

^d^10 (2%) missing data.

^e^3 (0.6%) missing data.

^f^32 (6.5%) missing data.

^g^70 (14.2%) missing data.

### Relationships Among Study Variables and WAI as a Function of Age

Correlation analyses are presented in Table 6. With respect to *anthropometric* measures, there was a negative association between waist circumference, hip circumference (but not waist-to-hip ratio), BMI, and work ability. This association was held for the entire sample and young adults but not in the older groups. *Cardiovascular* parameters, including blood pressure and heart rate at rest and after cycle ergometry, and indices of cardiovascular fitness in the Physical Work Capacity-130 test were positively associated with work ability in the older group, whereas self-reported weekly physical activity was positively associated with work ability only in young adults. In addition, hemoglobin, which indicates oxygen saturation and is considered an indicator of cardiovascular fitness, showed a positive correlation with work ability in the whole sample and even more so in older participants. No correlations with work ability were found for *metabolic* parameters. The same was true for selected *immunological* parameters such as the number of B cells, T cells and natural killer cells, monocytes, and granulocytes in the whole sample. However, a negative relationship was found between the number of B and T cells and work ability in older participants. A negative correlation with work ability was also found for immunological age (approximated by lymphocyte subsets using Principal Component Regression) and the CD4/CD8 ratio.

Regarding *personality* factors, an age-independent negative relationship was found between emotional instability (neuroticism) and work ability, and positive relationships were found between extraversion, conscientiousness, self-control, and work ability. Moreover, the early riser chronotype was positively associated with work ability, at least in young and older individuals. For the main *cognitive* functions assessed by neuropsychological tests, small positive associations with work ability were found for nearly all functions in the total sample only. The assessment of cognitive failure in daily living was negatively correlated with work ability in the entire sample and in each age group separately.

Substantial negative correlations with work ability were found for the following different types of *stress and stress-related health consequences*: acute stress (Psychosocial Stress Questionnaire–20); chronic stress (Trier Inventory of Chronic Stress); changes in stress resilience because of stress in childhood (Childhood Trauma Questionnaire); or perceived stress reactivity to immediate, intense, and long-lasting stress (Perceived Stress Reactivity Scale). Higher influence at work and control over work processes were positively associated with work ability. Similarly, time for recovery, ability to relax after work, and commitment at work reflect positive factors for work ability. In contrast, high emotional demands, high self-control at work, and emotional dissonance at work were clearly related to lower work ability. Negative associations with work ability were also found for work and social overload, pressure to perform, job dissatisfaction, job demands, lack of social recognition, social tension or isolation, and chronic worry. Substantial negative and age-independent correlations were observed between depressive symptoms (Beck Depression Inventory) or burnout (Maslach Burnout Inventory) and work ability.

The final correlation analysis was performed to determine *quality of life*. An overall positive relationship was found between work ability and physical, psychological, social, environmental, and global quality of life.

**Table 6:** Correlation analyses between the Work Ability Index total score and the anthropometric, cardiovascular, metabolic, immunological, cognitive, personality, occupational, and quality of life variables for the whole sample and separately for young, middle-aged, and older employees.

Variable			Correlation coefficient in the total sample		Correlation coefficient in the young group		Correlation coefficient in the middle-aged group		Correlation coefficient in the older group
Age (years)			*−0.241^a,b^*		N/A^c^		N/A		N/A
**Anthropometric variables**									
	Weight (kg)	−0.084		−0.139		−0.015		0.021	
	Waist size (cm)	*−0.132^d^*		*−0.196^d^*		0.010		−0.041	
	Hip size (cm)	*−0.171^d^*		*−0.187^d^*		−0.086		−0.087	
	WHR^e^	−0.006		−0.107		*0.210^d^*		0.067	
	BMI (kg/m^2^)	*−0.176^a^*		*−0.232^d^*		−0.091		−0.090	
**Cardiovascular variables**									
	DBP^f^ rest (mm Hg)	−0.041		−0.117		0.034		*0.180^g^*	
	SBP^h^ rest (mm Hg)	−0.066		*−0.159^g^*		0.086		*0.177^g^*	
	HR^i^ rest (bpm)	*−0.093^g^*		*−0.171^g^*		−0.031		−0.103	
	DBP max (mm Hg)	*0.115^g^*		−0.013		0.132		*0.245^d^*	
	SBP max (mm Hg)	0.087		−0.057		0.107		*0.263^d^*	
	HR max (bpm)	*0.126^d^*		−0.048		0.123		*0.270^d^*	
	QRS^j^ (ms)	*0.130^d^*		0.103		0.135		−0.069	
	PWC-130^k^ (W/kg)	*0.151^d^*		0.132		0.115		*0.232^d^*	
	PWC-130 max (W)	*0.143^d^*		0.060		*0.164^g^*		*0.278^d^*	
	Hb^l^	*0.119^d^*		0.117		0.123		*0.232^d^*	
	Activity (min/week)	*0.103^g^*		*0.244^d^*		0.104		0.056	
**Metabolic variables**									
	Creatinine	0.071		0.064		0.083		0.168	
	Cholesterol total	−0.057		−0.051		0.035		0.105	
	HDL^m^	−0.058		−0.018		−0.076		0.052	
	LDL^n^	−0.050		−0.073		0.056		0.072	
	Triglycerides	−0.015		−0.077		0.088		−0.019	
	CRP^o^	−0.048		−0.033		−0.075		−0.029	
**Immunological variables**									
	Immune age	*−0.128^d^*		−0.010		−0.049		−0.070	
	CD4/CD8 T cells	*−0.119^d^*		−0.088		−0.022		−0.003	
	CD4 memory/naive	*−0.133^d^*		−0.152		−0.098		−0.047	
	CD8 memory/naive	*−0.127^d^*		−0.016		0.013		−0.050	
	B cells/µl	−0.051		−0.056		−0.033		*−0.189^g^*	
	T cells/µl	−0.051		−0.067		0.005		*−0.217^g^*	
	NK^p^ cells/µl	−0.057		−0.090		0.011		−0.086	
	NK T cells/µl	−0.018		−0.142		0.037		0.035	
	Monocytes/µl	0.033		0.062		0.020		0.045	
	Granulocytes/µl	−0.004		−0.050		−0.057		0.108	
**Personality variables**									
	Emotional instability	*−0.433^a^*		*−0.453^a^*		*−0.508^a^*		*−0.483^a^*	
	Extraversion	*0.265^a^*		*0.157^g^*		*0.342^a^*		*0.278^d^*	
	Openness	0.018		−0.063		0.039		−0.002	
	Agreeableness	*0.157^d^*		0.095		*0.190^g^*		0.149	
	Conscientiousness	*0.159^d^*		*0.202^d^*		*0.160^g^*		*0.265^d^*	
	Self-Control	*0.186^d^*		*0.165^g^*		*0.286^a^*		*0.211^d^*	
	GRIT: Perseverance of effort	0.091		*0.184^g^*		0.147		0.108	
	DMEQ^q^: Chronotype	0.063		*0.207^d^*		0.021		*0.260^d^*	
**Cognitive variables**									
	Digit span total: memory	*0.102^g^*		0.107		0.071		0.047	
	Word fluency: verbal flexibility	−0.022		*0.183^g^*		0.009		−0.165	
	D2: sustained attention	*0.184^d^*		0.073		*0.149^g^*		0.021	
	MWT-B^r^: crystalized intelligence	*−0.119^d^*		0.039		−0.125		0.034	
	VLMT^s^: verbal learning span	*0.113^a^*		−0.009		0.131		−0.059	
	VLMT: delayed retrieval	0.031		−0.065		0.031		−0.042	
	Stroop: interference processing	*−0.153^d^*		−0.097		−0.096		−0.032	
	TMT^t^ B-A: cognitive flexibility	−0.046		−0.058		0.006		0.067	
	DST^u^: psychomotor processing	*0.143^d^*		0.116		0.050		−0.066	
	LPS^v^ 3: logical reasoning	*0.133^d^*		0.013		0.101		−0.023	
	LPS7: mental rotation	*0.095^g^*		0.114		0.085		−0.039	
	CFQ^w^: cognitive failures	*−0.271^a^*		*−0.261^d^*		*−0.421^a^*		*−0.187^g^*	
**Occupational stress- and job-related variables**									
	BDI^x^: depressive symptoms	*−0.517^a^*		*−0.518^a^*		*−0.589^a^*		*−0.486^a^*	
	MBI^y^: burnout symptoms	*−0.462^a^*		*−0.423^a^*		*−0.541^a^*		*−0.530^a^*	
	OLBI^z^: emotional exhaustion	*0.114^i^*		0.139		0.079		*0.220^g^*	
	CTQ^a1^: childhood trauma	*−0.248^a^*		−0.117		*−0.325^a^*		*−0.235^a^*	
	Job control	*0.195^a^*		*0.241^d^*		*0.200^d^*		*0.247^d^*	
	Self-control at work	*−0.243^a^*		*−0.225^a^*		*−0.308^a^*		*−0.180^g^*	
	Psychosocial Stress Questionnaire–20: psychosocial stress	*−0.469^a^*		*−0.441^a^*		*−0.528^a^*		*−0.490^a^*	
	Perceived Stress Reactivity Scale: stress reactivity	*−0.319^a^*		*−0.270^a^*		*−0.407^a^*		*−0.346^a^*	
	Emotional dissonance	*−0.277^a^*		*−0.238^g^*		*−0.332^a^*		*−0.287^g^*	
	Relaxation	*0.136^i^*		0.093		*0.186^d^*		*0.182^d^*	
	Exhaustion	*−0.416^a^*		*−0.304^a^*		*−0.526^a^*		*−0.525^a^*	
	Recovery	*0.319^a^*		*0.219^g^*		*0.383^a^*		*−0.419^a^*	
	Commitment	*0.177^g^*		0.132		*0.256^a^*		*0.251^g^*	
	Work overload	*−0.310^d^*		*−0.326^a^*		*−0.355^a^*		*−0.357^a^*	
	Social overload	*−0.223^d^*		*−0.236^g^*		*−0.163^d^*		*−0.193^d^*	
	Pressure to succeed	*−0.121^g^*		*−0.207^g^*		*−0.145^d^*		*−0.059^a^*	
	Work discontent	*−0.357>^a^*		*−0.295^a^*		*−0.484^a^*		*−0.439^a^*	
	Work demands	*−0.450^a^*		*−0.456^a^*		*−0.514^a^*		*−0.466^a^*	
	Lack of social recognition	*−0.379^a^*		*−0.337^a^*		*−0.456^a^*		*−0.267^g^*	
	Social tensions	*−0.310^a^*		−0.126		*−0.426^a^*		*−0.288^a^*	
	Social isolation	*−0.314^a^*		*−0.193^d^*		*−0.390^a^*		*−0.384^a^*	
	Chronic worrying	*−0.409^a^*		*−0.368^a^*		*−0.502^a^*		*−0.527^a^*	
	Short Scale of Chronic Stress: chronic stress	*−0.471^a^*		*−0.463^a^*		*−0.552^a^*		*−0.520^a^*	
**Quality of life**									
	Physical	*0.665^a^*		*0.643^a^*		*0.650^a^*		*0.730^a^*	
	Psychological	*0.472^a^*		*0.427^a^*		*0.567^a^*		*0.521^a^*	
	Social	*0.299^a^*		*0.252^a^*		*0.296^a^*		*0.290^a^*	
	Environmental	*0.313^a^*		*0.377^a^*		*0.380^a^*		*0.406^a^*	
	Global quality of life	*0.515^a^*		*0.473^a^*		*0.530^a^*		*0.593^a^*	

^a^Significant at the *P*<.001 level.

^b^Italicized values are those with significant results.

^c^N/A: not applicable.

^d^Significant at the *P*<.01 level.

^e^WHR: waist-to-hip ratio.

^f^DBP: diastolic blood pressure.

^g^Significant at the *P*<.05 level.

^h^SBP: systolic blood pressure.

^i^HR: heart rate.

^j^QRS: QRS Complex of the electrocardiogram.

^k^PWC-130: Physical Work Capacity at 130 bpm.

^l^Hb: hemoglobin.

^m^HDL: high-density lipoprotein.

^n^LDL: low-density lipoprotein.

^o^CRP: C-reactive protein

^p^NK cells: natural killer cells.

^q^D-MEQ: chronotype questionnaire.

^r^MWT-B: Multiple-Choice Vocabulary Test.

^s^VLMT: Verbal Learning and Memory Test.

^t^TMT: Trail Making Test.

^u^DST: Digit Symbol Test.

^v^LPS: Leistungs-Prüf-System (Performance Test System).

^w^CFQ: Cognitive Failures Questionnaire.

^x^BDI: Beck Depression Inventory.

^y^MBI: Maslach Burnout Inventory.

^z^OLBI: Oldenburg Burnout Inventory.

^a1^CTQ: Childhood Trauma Questionnaire.

### Work Ability Predictors in Each Domain

Results of multiple regression analyses in each domain are shown in Table 7. Overall, chronological age was consistently a strong negative predictor of work ability. For *anthropometric* variables, only BMI predicted work ability.

Among *cardiovascular* variables, those indicating physical fitness, such as diastolic blood pressure at rest, maximum heart rate during cycle ergometry, physical activity per week, and hemoglobin concentration, were found to be predictors of work ability. No influence of the selected blood *metabolic* variables on the work ability was observed. In contrast, the marker of *immunological* age and the CD4/CD8 T cells ratio as well as the concentration of monocytes were substantial predictors of work ability. Standardized *cognitive* tests did not explain work ability, whereas higher scores in the Cognitive Failures Questionnaire, indicating cognitive failures in daily life, explained lower work ability.

Of the *personality* traits, only emotional instability (neuroticism) negatively predicted work ability. *Occupational-, psychosocial-, and stress-related* factors were found to be important predictors of work ability, with a considerable predictive power of approximately 45.2%. Depressive symptoms, burnout, emotional exhaustion, job stress, and job demands negatively predicted work ability, whereas job attachment and pressure to succeed positively predicted work ability. Finally, the predictive power of *quality of life* was 51.2%, with physical and global quality of life being strong positive predictors of work ability.

**Table 7:** Standard linear multiple regression analyses including age in the anthropometric, cardiovascular, metabolic, immunological, cognitive, personality, occupational, stress-related, and quality of life domains with the Work Ability Index score as the dependent variable.

Model			Unstandardized coefficients, B		SE		Standardized coefficient (β)		*t* test (*df*)		*P* value
**Anthropometric variables^a^ (*R^2^*=.080; adjusted *R^2^*=.074; *F*_3,493_=14.15; *P*<.001; n=494)**											
	Age (years)	*−0.098^b^*		*0.02*		−*.223*		−*5.025 (490)*		*.001*	
	WHR^c^	3.95		2.8		.063		1.400 (490)		.16	
	BMI (kg/m^2^)	−*0.203*		*0.054*		−*.170*		−*3.736* (490)		*.001*	
**Cardiovascular variables (*R^2^*=.168; adjusted *R^2^*=.144; *F*_11,389_=6.94; *P*<.001; n=390)**											
	Age (years)	−*0.116*		*0.024*		−*.281*		−*4.920 (378)*		*.001*	
	SBP^d^ rest (mm Hg)	*0.061*		*0.030*		*.185*		*2.054 (378)*		*.04*	
	DBP^e^ rest (mm Hg)	−0.046		0.049		−.080		−.942 (378)		.35	
	HR^f^ rest (bpm)	−0.041		0.021		−.110		−1.915 (378)		.06	
	SBP max (mm Hg)	−0.023		0.017		−.091		−1.363 (378)		.17	
	DBP max (mm Hg)	0.030		0.020		.079		1.545 (378)		.12	
	HR max (bpm)	*0.099*		*0.032*		*.165*		*3.105 (378)*		*.002*	
	QRS^g^ (ms)	−0.012		0.022		−.031		−.568 (378)		.57	
	Hb^h^	*0.876*		*0.229*		*.211*		*3.824 (378)*		*.001*	
	PWC-130^i^ max (W)	0.002		0.011		.011		.141 (378)		.89	
	Physical activity (min/week)	*0.003*		*0.001*		*.120*		*2.384 (378)*		*.02*	
**Metabolic variables^j^ (*R^2^*=.069; adjusted *R^2^*=.058; *F*_6,488_=5.55; *P*<.001; n=489)**											
	Age (years)	−*0.111*		*0.021*		−*.255*		−*5.333 (482)*		*.001*	
	Creatinine	3.198		1.754		.084		1.823 (482)		.07	
	HDL^k^ (mmol/L)	−0.003		0.015		−.009		−.178 (482)		.86	
	LDL^l^ (mmol/L)	0.004		0.008		.023		.467 (482)		.64	
	Triglycerides (mmol/L)	−0.002		0.005		−.024		−.458 (482)		.65	
	CRP^m^	−0.096		0.102		−.042		−.940 (482)		.35	
**Immunological variables^n^ (*R^2^*=.042; adjusted *R^2^*=.025; *F*_8,485_=2.49; *P*=.012; n=486)**											
	Immune age	−*7.384*		*2.491*		−*.148*		−*2.964 (477)*		*.003*	
	CD4/CD8 T cells	−*1.235*		*0.497*		−*.119*		−*2.485 (477)*		*.01*	
	Number B cells/µL	−0.002		0.003		−.027		−.476 (477)		.63	
	Number T cells/µL	0		0		−.039		−.695 (477)		.49	
	Number NK^o^ cells/µL	−0.001		0.001		−.086		−1.452 (477)		.15	
	Number NK T cells/µL	0.001		0.003		.017		.350 (477)		.73	
	Number monocytes/µL	*0.006*		*0.003*		*.111*		*2.008 (477)*		*.045*	
	Number granulocytes/µL	0.004		0.005		.042		.848 (477)		.40	
**Cognitive variables (*R^2^*=.213; adjusted *R^2^*=.183; *F*_16,432_=7.03; *P*<.001; n=433)**											
	Age (years)	−*0.123*		*0.028*		−*.276*		−*4.346 (416)*		*.001*	
	MMSE^p^: general cognition	0.303		0.218		.067		1.386 (416)		.17	
	Digit span total: memory	0.056		0.078		.035		.719 (416)		.47	
	Word fluency: spoken	0.014		0.026		.034		.553 (416)		.58	
	Word fluency: written	−0.029		0.046		−.041		−.640 (416)		.52	
	D2: sustained attention	0.012		0.009		.075		1.262 (416)		.21	
	MWT-B^q^: crystalized intelligence	−0.114		0.104		−.062		−1.096 (416)		.27	
	VLMT^r^: learning span	0.040		0.044		.061		.910 (416)		.36	
	VLMT: verbal interference	−0.094		0.143		−.034		−.657 (416)		.51	
	VLMT: delayed retrieval	−0.191		0.119		−.093		−1.607 (416)		.11	
	Stroop: interference processing	−0.043		0.051		−.044		−.856 (416)		.39	
	TMT^s^ B-A: cognitive flexibility	0.005		0.013		.019		.387 (416)		.70	
	DST^t^: psychomotor processing	−0.023		0.031		−.048		−.744 (416)		.46	
	LPS^u^ 3: logical reasoning	0.013		0.059		.013		.221 (416)		.83	
	LPS7: mental rotation	0.011		0.037		.015		.293 (416)		.77	
	CFQ^v^: cognitive failures	−*0.171*		*0.023*		−*.332*		−*7.347* *(416)*		*.001*	
**Personality variables (*R^2^*=.343; adjusted *R^2^*=.329; *F*_9,393_=22.34; *P*<.001; n=394)**											
	Age (years)	−*0.155*		*0.020*		−*.348*		−*7.927* *(384)*		*.001*	
	Emotional instability	−*3.870*		*0.440*		−*.462*		−*8.804 (384)*		*.001*	
	Extraversion	0.729		0.522		.071		1.396 (384)		.16	
	Openness	−0.226		0.432		−.022		−.523 (384)		.60	
	Agreeableness	0.502		0.508		.043		.987 (384)		.32	
	Conscientiousness	0.272		0.570		.026		.477 (384)		.63	
	Self-control	0		0.029		−.001		−.014 (384)		.99	
	GRIT: perseverance of effort	−0.027		0.541		−.002		−.050 (384)		.96	
	D-MEQ^w^: chronotype	0.024		0.024		.044		.993 (384)		.32	
**Occupational stress control, burnout, and job control–related variables^y^ (*R^2^*=.480; adjusted *R^2^*=.452; *F*_23,459_=17.48; *P*<.001; n=460)**											
	Age (years)	−*0.150*		*0.017*		−*.350*		−*9.108 (435)*		*.001*	
	BDI^y^: depressive symptoms	−*0.140*		*0.045*		−*.158*		−*3.110 (435)*		*.002*	
	Maslach Burnout Inventory: burnout symptoms	−*0.079*		*0.027*		−*.168*		−*2.910 (435)*		*.004*	
	Oldenburg Burnout Inventory: emotional exhaustion	0.031		0.062		.018		.497 (435)		.62	
	Childhood Trauma Questionnaire: childhood trauma	−0.023		0.017		−.051		−1.350 (435)		.18	
	Job control	0.062		0.033		.072		1.848 (435)		.07	
	Self-control at work	−0.006		0.028		−.011		−.226 (435)		.82	
	Psychosocial Stress Questionnaire–20: psychosocial stress	−*0.063*		*0.021*		−*.215*		−*2.952 (435)*		*.003*	
	Perceived Stress Reactivity Scale: stress reactivity	0.024		0.030		.038		.802 (435)		.42	
	Emotional dissonance	−0.002		0.057		−.002		−.043 (435)		.97	
	Relaxation	−0.014		0.049		−.012		−.287 (435)		.77	
	Exhaustion	−*0.245*		*0.108*		−*.120*		−*2.261 (435)*		*.02*	
	Recovery	0.063		0.088		.036		.719 (435)		.47	
	Commitment	*0.071*		*0.031*		*.087*		*2.313 (435)*		*.02*	
	Work overload	0.074		0.053		.085		1.386 (435)		.17	
	Social overload	−0.061		0.057		−.056		−1.085 (435)		.28	
	Pressure to succeed	*0.092*		*0.042*		*.114*		*2.215 (435)*		*.03*	
	Work discontent	−0.042		0.050		−.043		−.838 (435)		.40	
	Demands from work	−*0.165*		*0.081*		−*.115*		−*2.033 (435)*		*.04*	
	Lack of social recognition	0.015		0.076		.010		.201 (435)		.84	
	Social tensions	0.047		0.061		.035		.764 (435)		.45	
	Social isolation	−0.036		0.055		−.029		−.655 (435)		.51	
	Chronic worrying	−0.067		0.092		−.044		−.731 (435)		.47	
**Quality of life (*R^2^*=.518; adjusted *R^2^*=.512; *F*_6,459_=86.77; *P*<.001; n=460)**											
	Age (years)	−*0.101*		*0.015*		−*.230*		−*6.750 (453)*		*.001*	
	Physical	*1.449*		*0.128*		*.538*		*11.295 (453)*		*.001*	
	Psychological	0.154		0.122		.064		1.267 (453)		.21	
	Social	0.128		0.070		.067		1.836 (453)		.07	
	Environmental	−0.079		0.119		−.028		−.666 (453)		.51	
	Global quality of life	*0.221*		*0.092*		*.115*		*2.409 (453)*		*.02*	

^a^Collinearity was found between BMI and weight, waist size and WHR, and hip size and WHR. Thus, weight, waist circumference, and hip size were excluded from analysis.

^b^Italicized values are those with significant results.

^c^WHR: waist-to-hip ratio.

^d^SBP: systolic blood pressure.

^e^DBP: diastolic blood pressure.

^f^HR: heart rate.

^g^QRS: QRS Complex of the electrocardiogram.

^h^Hb: hemoglobin.

^i^PWC-130: Physical Work Capacity at 130 bpm.

^j^Ammonia was excluded from the analysis because more than 50% of the data were missing. The total cholesterol score was excluded because of collinearity with HDL cholesterol.

^k^HDL: high-density lipoprotein.

^l^LDL: low-density lipoprotein.

^m^CRP: C-reactive protein.

^n^Hair cortisol was excluded due to missing data (≥50%), age was excluded from the analysis owing to the inclusion of the composite variable immune age and substantial collinearity between both variables.

^o^NK: natural killer.

^p^MMSE: Mini Mental State Examination.

^q^MWT-B: Multiple-Choice Vocabulary Test.

^r^VLMT: Verbal Learning and Memory Test.

^s^TMT: Trial Making Test.

^t^DST: Digital Symbol Test.

^u^LPS: Leistungs-Prüf-System (Performance Test System).

^v^CFQ: Cognitive Failures Questionnaire.

^w^D-MEQ: chronotype questionnaire.

^x^The short scale of chronic stress was excluded because of collinearity with other dimensions of the TICS.

^y^BDI: Beck Depression Inventory.

### Essential Predictors of Work Ability

In the final regression model presented in Table 8, only the predictors that demonstrated the largest explanatory impact on work ability in each domain were considered. The model showed a reasonable predictive power of 51.6% and extracted 8 essential predictors of work ability: age, maximum heart rate, hemoglobin, depressive and burnout symptoms, commitment, pressure to succeed, and physical quality of life.

**Table 8:** Standard linear multiple regression analysis including significant predictors extracted in the low-level regression analyses from the 8 domains with Work Ability Index as the dependent variable^a^.

Model^b^		Unstandardized coefficients, B	SE	Standardized coefficient (β)	*t* test (*df*)	*P* value
**Essential predictors (*R^2^*=.525; adjusted *R^2^*=.516; F_13,376_=51.05; *P*<.001; n=377)**						
	Age (years)	−*0.102*^c^	*0.015*	−*.256*	−*6.643 (368)*	*.001*
	HR^d^ max	*0.053*	*0.023*	*.089*	*2.335 (368)*	*.02*
	Hb^e^	*0.398*	*0.150*	*.099*	*2.642 (368)*	*.009*
	BDI^f^	−*0.181*	*0.042*	−*.204*	−*4.307 (368)*	*.001*
	MBI^g^	−*0.047*	*0.021*	−*.106*	−*2.301 (368)*	*.02*
	Commitment	*0.073*	*0.028*	*.096*	*2.581 (368)*	*.01*
	Pressure to succeed	*0.066*	*0.029*	*.088*	*2.264 (368)*	*.02*
	Physical quality of life	*1.270*	*0.127*	*.457*	*1.019 (368)*	*.001*

^a^The method used was backward elimination. Only significant predictors are displayed. In total, 11 multivariate outliers assessed by the Mahalanobis distance were identified and removed.

^b^*R*^2^=.525, adjusted *R*^2^=.516; *F*_13,376_=51.05; *P<*.001; n=377.

^c^Italicized values are those with significant results.

^d^HR: heart rate.

^e^Hb: hemoglobin.

^f^BDI: Beck Depression Inventory.

^g^MBI: Maslach Burnout Inventory.

## Discussion

### Principal Findings

The aim of this study was to systematically analyze several endogenous (biological) and exogenous (environmental) factors that may contribute to the work ability in different age groups of the working population in 494 healthy adults. Overall, the sample consisting of individuals from heterogeneous work sectors (industry, craft, service, and education) showed good work ability (39 out of possible 49 points), which was reported to decrease with chronological age [5,24-27]. Sociodemographic factors such as marital status, raising underage children, housing situation, care of relatives, nutrition, and use of stimulants such as smoking or alcohol consumption were not related to work ability [14,28]. The same was true for some work-related factors such as work amount, type of employment contract, commuting to work, or shift work, confirming previous reports [29,30] but in contradiction to other result, showing lower work ability with shift work [31-33]. The absence of the latter association may be due to the low proportion of shift workers in our sample. Education and the frequency of the use of a foreign language, which are proxies for socioeconomic status and type of work, were positively associated with work ability. Employees with lower levels of education tend to work in physically demanding jobs, whereas employees with higher levels of education tend to work in mentally demanding jobs [14]. This was also confirmed by the fact that work ability was lower in the group with repetitive work than in those working flexibly. An important finding was that sleep problems were associated with lower work ability, which is consistent with previous reports [28,34]. On the other hand, social components, such as the number of close friends and the frequency of friendship meetings, were associated with increased work ability, which may indicate that a satisfactory private social environment plays an important role in work ability [6,16]. In line with this, social support has been considered as one of the key resources for coping with work demands [35]. The presence of a pet in the family was associated with lower work ability, which is surprising, considering that pet ownership is usually related to positive health outcomes [36]. In contrast to physical activity and social contacts, computer or mobile phone use was not the preferred free-time activity in this cohort. Although time of computer or smartphone use during leisure time had no effect on work ability, individuals who watched ≥3 hours of television per day had lower work ability, which appears to be associated with lower levels of education.

The results were largely confirmed by regression models conducted to identify the specific individual determinants affecting work ability in each domain. In general, chronological age was consistently a strong negative predictor of work ability [5]. Results in the anthropometric domain indicate that BMI predicts work ability, suggesting that overweight or obesity is a risk factor for low work ability [34,37-39]. Objectively measured cardiovascular variables indicative of physical fitness, such as resting diastolic blood pressure, maximum heart rate on cycle ergometry, and hemoglobin concentration, indicating oxygen-carrying capacity, were also found to be positive predictors of work ability and were consistent with self-reported weekly physical activity. These results emphasize the importance of cardiovascular fitness for the physical health status reflected in work ability [14,15,40].

No effect of the selected blood metabolic variables on the WAI was observed. However, immunological age [41] and the CD4/CD8 ratio, which are associated with age-related changes in immune responses [42], appear to be negative predictors of work ability, whereas the concentration of monocytes in the blood, which are necessary to find and destroy viruses, bacteria, fungi, and protozoa and to eliminate infected cells, positively predicted work ability.

As expected, associations between work ability and personality variables were not prevalent in any particular age group: emotional instability (neuroticism) was generally negatively associated with work ability, suggesting that individuals with high levels of anxiety, worry, fear, guilt, and depressed mood are likely to respond worse to stressors, which may lead to lower work ability. In contrast, extroverted individuals, who are characterized by outgoing, talkative, and energetic behavior, may be more resilient and successful in coping with work-related problems or in selecting jobs that better match their abilities than emotionally instable individuals. We also observed a positive and age-independent relationship between conscientiousness or self-control and work ability [43]. Moreover, the early riser chronotype was positively associated with higher work ability. Regression modeling in this domain revealed that only neuroticism explained work ability.

The most consistent and age-independent negative associations with work ability were observed for factors related to psychosocial work demands and work stress and their potential long-term mental health consequences. Substantial negative associations with work ability were found for several types of stress and for work and social overload, job dissatisfaction, lack of social recognition, social tension or isolation, and chronic worry. High emotional demands, high self-control at work, and emotional dissonance were clearly associated with lower work ability (Table 6). Several of these factors reflect an imbalance between demands and available resources and may contribute to long-lasting symptoms of emotional exhaustion, burnout, and depressive symptoms, confirmed by the substantial negative association with work ability [44-46]. Regression models showed that depressive symptoms, burnout, emotional exhaustion, job stress, and job demands negatively predicted work ability, whereas job attachment and pressure to succeed positively predicted work ability [28,47], which is consistent with the observed positive effects of higher influence at work, control over work processes, and commitment at work. Thus, the negative and positive determinants appear to be 2 sides of the same coin in preventing poor work ability and promoting high work ability [48].

Cognitive functions assessed by neuropsychological tests showed a small positive association with work ability only in the whole sample, but the regression model did not reveal any substantial predictor. An important exception was the assessment of cognitive failures in daily living. This result suggests that frequent attention and memory lapses in daily living predict lower work ability regardless of age, confirming previous observations in nurses [37]. Finally, an overall positive relationship was found between work ability and physical, psychological, social, environmental, and global quality of life. The regression model revealed that physical and global quality of life were predictors of work ability. Together with the role of social relations outside the workplace discussed earlier, this finding indicates that not only work-related circumstances but also general life satisfaction is crucial for high work ability [5,6,16].

The final regression model, which included the determinants with the strongest predictability, showed that 8 out of the 80 predictors explained approximately 52.5% of the variance in work ability. In addition to chronological age, depressive and burnout symptoms had the strongest negative impact on work ability. Moreover, maximum heart rate and hemoglobin concentration as indices of physical fitness, as well as commitment to the company, pressure to succeed, and physical quality of life were factors with substantial positive predictive values for work ability. This result confirms that work ability is a complex and multifactorial concept that consists of determinants with different weights.

### Limitations and Future Challenges

Some potential weaknesses and strengths of the study need to be acknowledged. First, we performed a series of multiple regression analyses rather than a hierarchical regression analysis that include all predictors at once. This strategy was chosen to reduce the impact of missing data, as we used a complete-case analysis (listwise deletion). Second, we used a multitude of potential predictors grouped in theoretically based domains. The separate regression analyses resulted in an increase in power because the sample size in each analysis was at least 10 times larger than the number of predictors [15]. Third, for statistical power, we did not distinguish by sex, and the influence of these variables on work ability (such as work amount) could be different in male, female, or nonbinary employees.

Fourth, there may be some bias in the study population that could limit the generalizability of the results. The overall WAI score was 39, indicating a relatively healthy cohort, both from a physical and mental perspective. This could explain why diet has no influence on work ability, as most people eat fruits and vegetables almost every day and eat very few fast foods. Fifth, and somewhat related to the first point, the percentage of participants with a university degree (216/494, 43.7%) was higher than that in the general population in Germany (31.1%) in the age range of 25 to 64 years [51]. One might expect that a higher level of education would be associated with higher WAI scores, but this was not the case here. The mean WAI score of participants with a university degree was similar to that of individuals with an elementary school degree and even lower than that of individuals with secondary or high school degrees. This suggests that a university degree is not necessarily associated with high work ability. Self-selection bias related to education appears to be a general problem in studies based on the participants’ voluntary participation. Participants with an academic education might be more interested in participating in health-related studies. In other words, voluntary contribution prevents the gathering of a fully representative sample. However, as stated in the research protocol [17], the sample did not differ from the general population with respect to several relevant parameters such as age distribution, genetics, biological markers, or cognitive abilities. Sixth, despite the various sociodemographic aspects collected in the study, questions regarding financial security that may play a role in work ability were not included in the questionnaires.

Finally, it should also be noted that this study is a cross-sectional analysis of data from the *Dortmund Vital Study*, whereas a longitudinal design is the more informative and conclusive approach. Follow-up data are currently collected and will be published in few years.

The relevant strength of the study is that the selection of determinants was blinded to the WAI measurement. Furthermore, the selection of various biological and environmental parameters reflects the multifactorial nature of work ability. It is also important to note that the data were collected before the COVID-19 outbreak and, therefore, were not affected by the pandemic, which can have a large impact on middle-aged or older individuals [52]. Follow-up measures will reveal the potential impact of the COVID-19 pandemic, health issues, and individual living situations over the past 5 years on work ability.

### Promotion of Study Sustainability and Recommendations for Occupational Safety and Health

Given the present and previous findings regarding aspects affecting work ability, it seems important to raise awareness of protective factors that may enhance work ability among policy makers, employers, occupational safety and health (OSH) personnel in different occupational settings, and insurers and to emphasize the need for lifelong maintenance or even improvement of work ability in both personal and occupational contexts. As a first step, a set of recommendations and guidelines should be developed that consider the role of age, psychosocial stress, work-life balance, regular physical activity to improve cardiovascular capacity, sleep quality, immune status, lifelong learning, social contacts, and other critical factors for work ability. These guidelines can be disseminated through workshops, ideally organized during working hours, to inform workers and multipliers about the importance of private and work-related factors in preventing poor work ability and promoting high work ability. These recommendations may include information on the following: (1) stress and its negative influence on work ability; (2) the role of personal thinking style in stress perception; (3) the role of socially supportive behavior by colleagues and supervisors in the workplace context to reduce the impact of stressors; (4) the role of physical activity on stress, cardiovascular, and immune functions, as well as mental health; (5) the role of mental activity in maintaining cognitive function; and (6) the role of certain working conditions, such as monotonous work or intellectually low-demand work on work ability.

As a second step, targeted training programs could be offered by employers or occupational health professionals (eg, in collaboration with health insurance companies) to promote and train aspects of physical, mental, and emotional competencies. As a third step, employers and OSH personnel can use recommendations to improve work-related physical and psychosocial conditions.

A possible economic solution to improve work ability is to use an interactive system that recommends various human-centered training units. A recently completed project funded by the EU Commission (sustAge, sustainable work through technology-assisted enhancement of cognitive abilities of older employees) [53,54] aimed to develop an intelligent system that supports the cognitive, emotional, and physical conditions of older workers in production. This interactive system was designed to detect stress, fatigue, or decreased performance using sensor and speech recognition algorithms. If such conditions occur, the system recommends alternative work activities (eg, workplace rotation) or microbreaks with some units of cognitive training, or in the case of stress or fatigue, stress-reducing sessions such as progressive muscle relaxation can be performed. In the long term, such a solution would improve OSH and increase physical, cognitive, and emotional functioning; job satisfaction; and, consequently, work ability in the middle-aged and older workforce, which may prevent early retirement.

### Conclusions

In this study, several objective and subjective factors were identified to predict work ability as a function of age. Using the measures of our study can support the assessment of work ability in its complexity. Such a well-elaborated assessment of work ability can contribute to the effective management of OSH, sustainable work, and healthy aging by reducing health risks that lead to long-term sick leave or early retirement. Although chronological age, metabolic status, or personality traits reflect stable factors, other determinants related to physical fitness or psychosocial situations at work leading to stress may be modified. The extracted determinants provide OSH practitioners with tools that can be integrated into targeted physical, nutritional, cognitive, and stress-related preventive programs to improve current and future work ability in an aging society.

## Appendix

Multimedia Appendix 1 Supplementary material including methods (tests, questionnaires, and blood samples).

## Abbreviations

CD: cluster of differentiation
OSH: occupational safety and health
VIF: variance inflation factor
WAI: Work Ability Index

## Data Availability

The study data are available upon reasonable request from the authors (see the *Research Data Management* section in the study protocol by Gajewski et al [17]).
