# Supplementary material for: A Systematic Analysis of Biological, Sociodemographic, Psychosocial, and Lifestyle Factors Contributing to Work Ability Across the Working Life Span: Cross-sectional Study
Source: JMIR Form Res. 2023 May 19;7:e40818. doi: 10.2196/40818 (PMC10238961; doi:10.2196/40818)
Supplement: Multimedia Appendix 1 [file formative_v7i1e40818_app1.docx]

**Supplemental Appendix 1**

**Questionnaires**

The test battery included non-standardized and standardized questionnaires. Non-standardized questionnaires were administered to obtain sociodemographic and lifestyle variables (marital status, children, education, ability to speak foreign languages, type and history of employment, history of physical activity, nutrition, smoking, social activities, hobbies, digital media use, caregiving of family members, etc.). Standardized questionnaires measured depressive symptoms (Becks Depression Inventory [1]), personality traits (Big Five Personality traits, NEO-FFI, [2]), traumatic experiences in the childhood (Childhood Trauma Questionnaire, CTQ, [3]), chronotype (D-MEQ, [4]), cognitive failures in daily life (Cognitive Failure Questionnaire, CFQ, [5]), grit personality trait (Grit Scale, [6]), emotional dissonance [7], influence at work and job control [8], physical activity (Lüdenscheid Physical Activity Questionnaire, [9]), Burnout (Maslach Burnout Inventory, MBI-GS, [10], and Oldenburg Burnout Inventory (OLBI, [11]), Stress reactivity (Perceived Stress Reactivity Scale, PSRS, [12]), psychosocial stress (Psychosocial Stress Questionnaire, PSQ-20, [13]), self-control and self-control at work [14], chronic stress (Trier Inventory of Chronic Stress, TICS [15]), that consists of several domains: Work Overload, Social Overload, Pressure to Perform, Work Discontent, Demands from work, Lack of Social Recognition, Social Tensions, Social Isolation, Chronic Worrying, SSCS), and quality of life (WHOQoL-BREF, [16]).

**Neuropsychological assessment**

Details of the neuropsychological testing are described in [17]. Briefly, a wide range of cognitive functions was evaluated using standardized neuropsychological tests, measuring general cognitive status (Mini-Mental-State-Examination; MMSE; [18]), memory span and working memory (Digit-Span forward and backward from WAIS-III; [19]), semantic memory in written and oral version (Word-Fluency test; LPS, [20]), selective attention and attentional endurance (D2-R; [21]), crystallized intelligence and general knowledge (Multiple Choice Vocabulary Test; MWT-B; [22], different aspects of verbal memory like learning and memory retrieval (Verbal Learning and Memory Tests VLMT; [23]), psychomotor performance and speed of processing (Digit-Symbol-Test from WAIS-III; [19]), interference control and inhibition (Stroop-Test; [24]), task switching (Trail-Making-Test; TMT-A and TMT-B; [25]) and two further subtests of the performance testing system (LPS, [20]), measuring logical reasoning and spatial rotation.

**Measurement of physiological parameters**

*Physical fitness test*, *cardiovascular and anthropometric parameters*

Participants’ current physical performance was assessed with the physical work capacity (PWC-130) cycle test [26], using a bicycle ergometer. The aim of this test is to predict the absolute power output at a projected heart rate of 130 beats per minute. Relative power output is calculated by the power-to-weight ratio. In addition, pulse, electrocardiography (ECG), systolic, and diastolic blood pressure were recorded before and during ergometry. Anthropometric parameters like height, weight, waist-to-hip ratio, and Body Mass Index (BMI) were obtained from each participant.

*Blood samples and immunological analysis*

Analytical details related to blood sampling were described in [27]. Briefly, peripheral venous blood (80 mL) was collected from the participants in heparinized monovettes (Sarstedt, Nümbrecht, Germany) and a set of relative blood cell frequencies was determined by flow cytometry. Peripheral blood mononuclear cells (PBMC) were isolated by Ficoll density gradient centrifugation (PAN-Biotech, Aidenbach, Germany), and were stored at 170°C for up to 6 months until analysis. Four antibody panels were built to gain information on the general lymphocyte and monocyte subpopulations and to analyze the lymphocytes for NK/T cell ratio, CD4/CD8 T cell ratio, memory/naïve sub-populations of CD4+ and CD8+ T cells, and CD28^-^ T cells, which are all related to aging and senescence. All antibodies were individually titrated to determine the optimal dilution. We stained PBMC immediately after thawing and kept them on ice during the entire procedure. For each panel, we stained 0.2 x 10^6^ cells with the indicated antibody cocktails for 20 min in the dark at 4°C and afterwards washed them with FACS buffer (PBS/2% FCS). Cells were resuspended in FACS buffer and kept on ice until analysis at the same day on a BD LSRFortessa. Data were analyzed using the FlowJo software (FlowJo LLC, Ashland, OR, USA).

A metric of immune age (IMM-AGE) incorporating high-dimensional ‘omics’ technologies was approximated by lymphocyte subsets using Principal Component Regression. IMM-AGE describes the aging of a person’s immune status better than chronological age [28].

*Metabolic parameters*

From venous blood, the concentration of ammonia was extracted. Furthermore, concentration of triglycerides, cholesterol, high- and low-density lipoprotein cholesterol, glycosylated hemoglobin, C-reactive protein (CRP), and creatinine concentration were measured.

*Endocrine parameters*If possible, a hair sample was taken from which hair cortisol concentrations were measured as an index of long-term stress.

**References**

1. Beck AT, Steer RA, Brown GK. Manual for the Beck Depression Inventory-II. San Antonio, TX: Psychological Corporation; 1996.
2. Costa Jr PT, McCrae RR. Revised NEO Personality Inventory (NEO-PI-R) and NEO Five-Factor Investory (NEO-FFI) Professional Manual. Psychological Assesment Ressources, Inc; 1992.
3. Bernstein DP, Stein JA, Newcomb MD, Walker E, Pogge D, Ahluvalia T et al. Development and validation of a brief screening version of the Childhood Trauma Questionnaire. Child Abuse Negl 2003; 27(2): 169-190.
4. Griefahn B, Künemund C, Bröde P, Mehnert P. Zur Validität der deutschen Übersetzung des Morningness-Eveningness-Questionnaires von Horne und Östberg. Somnologie 2001; 5:71-80.
5. Broadbent DE, Cooper PF, FitzGerald P, Parkes KR. The cognitive failures questionnaire (CFQ) and its correlates. Br J Clin Psychol 1982; 21:1-16.
6. Duckworth AL, Quinn PD. Development and validation of the Short Grit Scale (GritS). J Pers Assess 2009; 91: 166-174.
7. Neubach B, Schmidt K-H. Selbstkontrolle als Arbeitsanforderung: Rekonzeptualisierung und Validierung eines Messinstruments [Self-control as job requirement: Reconceptualization and validation of a measuring instrument]. Z Arbeits Organisationspsychol 2006; 50:103–109. doi: 10.1026/0932-4089.50.2.103
8. Jackson PR, Wall TD, Martin R, Davids K. New measures of job control, cognitive demand, and production responsibility. J Appl Psychol 1993; 78(5):753-762. doi.org/10.1037/0021-9010.78.5.753
9. Höltke V, Jakob E. Lüdenscheider Aktivitätsfragebogen zum Risikofaktor Bewegungsmangel. Sportmedizin Hellersen, Lüdenscheid; 2002.
10. Schutte N, Toppinen S, Kalimo R, Schaufeli WB. The factorial validity of the Maslach Burnout Inventory - General Survey (MBI-GS) across occupational groups and nations. J Occup Org Psychol 2000; 73: 53-66.
11. Demerouti E, Bakker AB, Nachreiner F, Schaufeli WB. The job demands‐resources model of burnout. J Appl Psychol 2001; 86: 499‐512.
12. Schlotz W, Yim IS, Zoccola PM et al. The perceived stress reactivity scale: measurement invariance, stability, and validity in three countries. Psychol Assess 2011; 23:80–94. doi:10.1037/a0021148
13. Fliege H, Rose M, Arck P, Levenstein S, Klapp B. Validierung des Perceived Stress Questionnaire (PSQ) an einer deutschen Stichprobe. Diagnostica 2001; 47:147-157.
14. Neubach B, Schmidt K-H. Entwicklung und Validierung von Skalen zur Erfassung verschiedener Selbstkontrollanforderungen bei der Arbeit [Development and validation of scales assessing different job-related self-control demands]. Z Arbeitswiss 2007; 61:35-45.
15. Schulz P, Schlotz W, Becker P. Trierer Inventar zum chronischen Stress (TICS). Göttingen: Hogrefe; 2004.
16. World Health Organization. WHOQOL-BREF: Introduction, Administration, Scoring and Generic Version of the Assessment-Field Trial Version. WHO, Geneva; 1996.
17. Gajewski PD, Hanisch E, Falkenstein M, Thönes S, Wascher E. What does the n-back task measure as we get older? Relations between working-memory measures and other cognitive functions across the lifespan. Front Psychol 2018a; 9:2208.
18. Folstein MF, Folstein SE, Mc Hugh PR. Mini-mental state. A practical method for grading the cognitive state of patients for the clinican. J Psychiatr Res 1975; 12:189-198.
19. Wechsler D. WAIS-IIIR manual. New York: The Psychological Corporation, 1998.
20. Sturm W, Wilmes K, Horn W. LPS 50+ Leistungsprüfsystem für 50 bis 90-Jährige. Second Ed. Göttingen: Hogrefe; 2015.
21. Brickenkamp R, Schmidt-Atzert L, Liepmann D. d2-R: Test d2 - Revision. Hogrefe, Göttingen; 2010.
22. Lehrl S. Mehrfach-Wortwahl-Test (MWT). Medizinische Verlagsgesellschaft, Erlangen; 1995.
23. Helmstaedter C, Durwen HF. VLMT: verbaler Lern- und Merkfähigkeitstest. Ein praktikables und differenziertes Instrumentarium zur Prüfung der verbalen Gedächtnisleistungen. Schweiz Arch Neurol Psychiatr 1990; 141:21-30.
24. Stroop JR. Studies of interference in serial verbal reactions. J Exp Psychol 1935; 18: 643-662.
25. Reitan RM. Trail Making Test: Manual for Administration and Scoring. Reitan Neuropsychology Laboratory, Tucson, AZ; 1992.
26. Campbell, P. T., Katzmarzyk, P. T., Malina, R. M., Rao, D. C., Pérusse, L., & Bouchard, C. (2001). Prediction of physical activity and physical work capacity (PWC150) in young adulthood from childhood and adolescence with consideration of parental measures. American Journal of Human Biology, 13(2), 190–196. https://doi.org/10.1002/1520-6300(200102/03)13:2
27. Claus M, Dychus N, Ebel M, Damaschke J, Maydych V, Wolf OT, Kleinsorge T, Watzl C. Measuring the immune system: a comprehensive approach for the analysis of immune functions in humans. Arch Toxicol 2016; 90:2481-2495.
28. Alpert A, Pickman Y, Leipold M, Rosenberg-Hasson Y, Ji X, Gaujoux R, Rabani H et al. A clinically meaningful metric of immune age derived from high-dimensional longitudinal monitoring. Nat Med 2019; 25:487-495.
